# Design and synthesis of proton-dopable organic semiconductors†

**DOI:** 10.1039/d2ra00216g

**Published:** 2022-02-28

**Authors:** Chenzhu Yin, Masakazu Mukaida, Shohei Horike, Kazuhiro Kirihara, Shogo Yamane, Zhenya Zhang, Qingshuo Wei

**Affiliations:** Graduate School of Life and Environmental Sciences, University of Tsukuba 1-1-1, Tennodai Tsukuba Ibaraki 305-8572 Japan; Nanomaterials Research Institute, Department of Materials and Chemistry, National Institute of Advanced Industrial Science and Technology 1-1-1 Higashi Tsukuba Ibaraki 305-8565 Japan qingshuo.wei@aist.go.jp; Research Institute for Sustainable Chemistry, Department of Materials and Chemistry, National Institute of Advanced Industrial Science and Technology (AIST) 1-1-1 Higashi Tsukuba Ibaraki 305-8565 Japan; Department of Chemical Science and Engineering, Graduate School of Engineering, Kobe University 1-1 Rokkodai-cho Kobe 657-8501 Japan

## Abstract

This paper shows how protonated 3,4-ethylene dioxythiophene moieties can be used as an end group to make organic conductors. An organic semiconductor 2,5-bis(5-(2,3-dihydrothieno[3,4-*b*][1,4]dioxin-5-yl)-3-dodecylthiophen-2-yl)thieno[3,2-*b*]thiophene is designed and synthesized. This molecule could be doped by protonic acid in both solution and solid-state, resulting in a broad absorption in the near-infrared range corresponding to polaron and bipolaron absorption. Electrical conductivity of *ca.* 0.1 S cm^−1^ was obtained at 100 °C (to avoid the water uptake by the acid). The adducts with protons bound at the end-thiophene α-position were confirmed by ^1^H Nuclear Magnetic Resonance spectra.

## Abstract 2

The use of protonated 3,4-ethylenedioxythiophene moieties as an end group could be a promising approach to prepare organic conductors.

## Graphical abstract

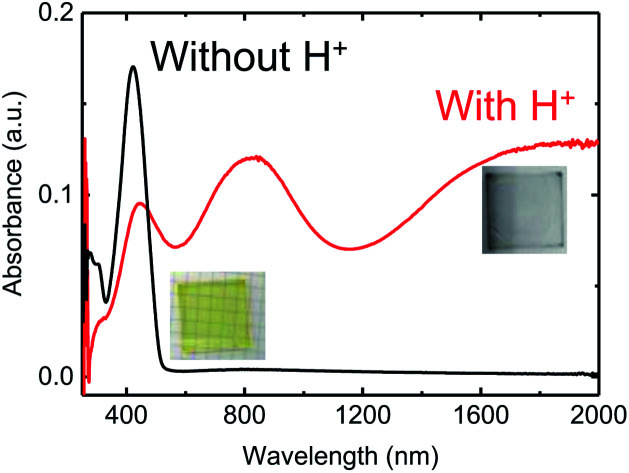

## Introduction

Researchers have put a lot of effort into polymer research after Shirakawa, Heeger, and Macdiarmid discovered that iodine treatment could increase the conductivity of polyacetylene by about 5 orders of magnitude from halogen uptake.^1^ Conducting polymers have successfully been used as active materials in various electronic devices, such as organic light-emitting diodes (OLEDs), organic solar cells (OSCs), thin-film transistors (TFTs), thermoelectrics, batteries, and biosensors, according to pioneering research.^2,3^

Generally, two distinct doping mechanisms exist in p-type conducting polymers.^4^ The first is oxidative doping, a chemical oxidation reaction, based on charge transfer from the host to the dopant. Oxidative doping mechanisms are used in conducting polymers doped with I_2_, FeCl_3_, SbCl_5_, and tetracyanoquinodimethane (TCNQ).^5,6^ The second is protonic doping, which has mostly been studied in nitrogen-containing organic semiconductors, *e.g.*, polyaniline.^7,8^ The charge delocalizes within the polymer chains when protons interact at the nitrogen sites, resulting in electronic conductivity.

PEDOT poly(3,4-ethylenedioxythiophene) has gained the most attention among the various forms of conducting polymers, both academically and in practical applications.^9^ High electrical conduction was achieved with the solution-processable PEDOT film (>4000 S cm^−1^),^10–12^ and single-crystal PEDOT nanowires synthesized under geometrically-confined conditions produced electrical conduction of up to 8000 S cm^−1^.^13^ PEDOT has no nitrogen sites and is known to be synthesized using Fe^3+^ or peroxodisulfate as an oxidant. As a result, it is believed that oxidizing agents are used to dope PEDOT.^14^ Aleshin *et al.*, on the other hand, reported that the conductivity and activation energy of PEDOT are sensitive to the pH value.^15^ Several pioneering groups recently, proposed that the acido-basic treatment could be used to control the thermoelectric performance of PEDOT.^16,17^ We proposed that PEDOT could be doped by proton and the protonic doping/de-doping is reversible.^18^ According to DFT calculations, the adducts with protons bound at the end-thiophene α-position are the most desirable. Therefore, it is natural to consider that the use of protonated EDOT moieties, as an end group in the preparation of organic conductors is a promising approach toward synthesizing conductive organic materials.

## Results and discussion

The organic semiconductor 2,5-bis(5-(2,3-dihydrothieno[3,4-*b*][1,4]dioxin-5-yl)-3-dodecylthiophen-2-yl)thieno[3,2-*b*]thiophene (BDTTT) was designed and synthesized in this study. This molecular could be doped by protons both in solution and in solid film. The largest advance of this molecule is a relatively good solubility in different solvents such as toluene and chloroform, and we were able to confirm the doping site using Nuclear Magnetic Resonance (NMR) analysis.

**Scheme 1:**
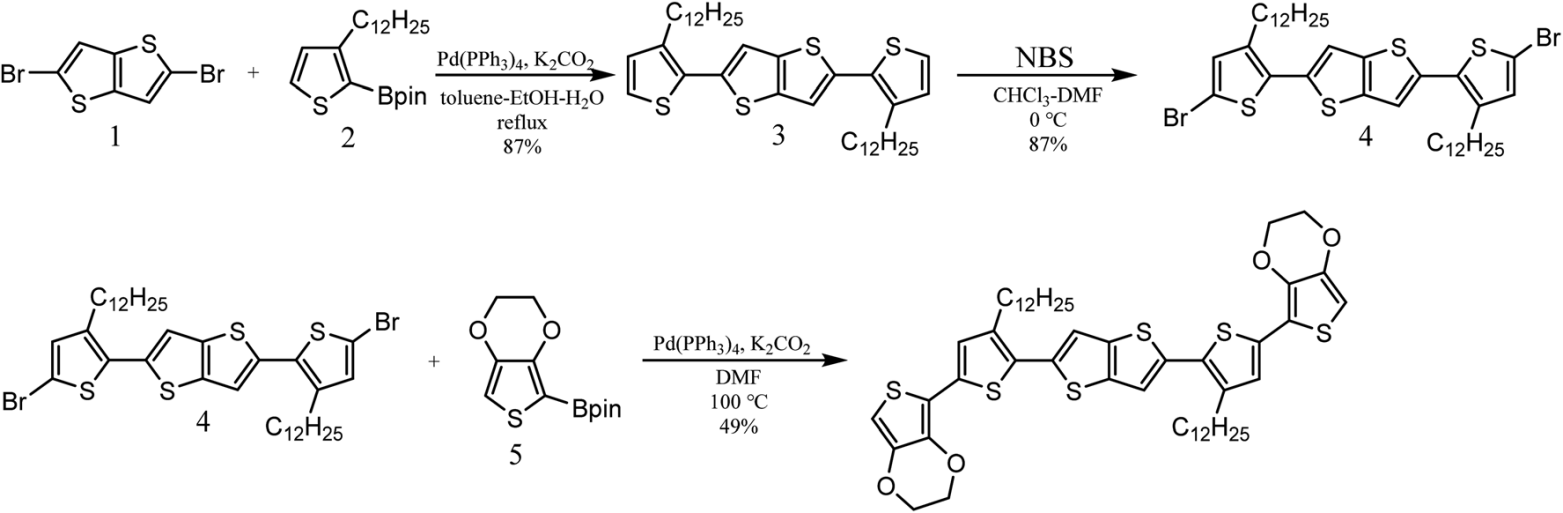
Synthesis route of BDTTT.

The synthetic route for the target molecule BDTTT is shown in Scheme 1. 2,5-Bis(3-dodecylthiophen-2-yl)thieno[3,2-*b*]thiophene (3) was synthesized by the palladium catalyzed cross coupling reaction of 2,5-dibromothieno[3,2-*b*]thiophene (1) with 3-dodecyl-2-(4,4,5,5-tetramethyl-1,3,2-dioxaborolan-2-yl)thiophene (2).^19^ 2,5-Bis(5-bromo-3-dodecylthiophen-2-yl)thieno[3,2-*b*]thiophene (4) was obtained by bromination of 3 with *N*-bromosuccinimide (NBS). The final product BDTTT was synthesized by the palladium catalyzed cross coupling reaction of (4) with 2-(2,3-dihydrothieno[3,4-*b*][1,4]dioxin-5-yl)-4,4,5,5-tetramethyl-1,3,2-dioxaborolane (5).^20^ The total yield from (1) is about 37%. BDTTT shows good solubility in different solvents including toluene, chloroform and tetrahydrofuran.

**Fig. 1:**
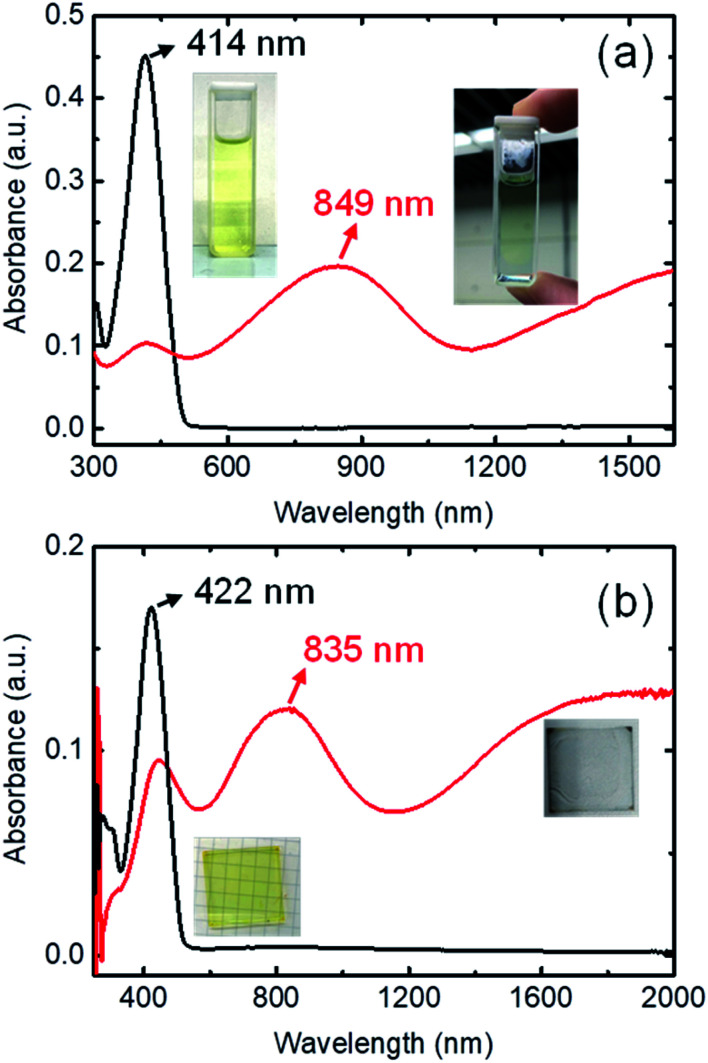
(a) Absorption spectra of 0.125 mM BDTTT in toluene without the addition of CF_3_COOH (black line), and with 0.375 M of CF_3_COOH (red line); Inset: photographs of toluene solutions of BDTTT with (right) and without (left) the addition of CF_3_COOH. (b) Absorption spectra of BDTTT film (black line), and DDBSA-BDTTT film (red line). Inset: photographs of the films.

The ultraviolet-visible-near-infrared (UV-Vis-NIR) absorption spectrum of 0.125 mM BDTTT in toluene solution is shown in Fig. 1a. The π–π* transition is responsible for a dominated absorption peak at 414 nm (approximately 3.0 eV) in pristine BDTTT solution. No absorption could be observed in the area of ≥500 nm. The color of the solution significantly changed from light yellow to light blue when 0.375 M trifluoroacetic acid (CF_3_COOH) was added to the solution. The UV-Vis-NIR absorption spectrum for the solution with lower acid concentration is shown in Fig. S1.† At high doping levels corresponding to the combination of polaronic and bipolaronic charge carriers, the intensity of π–π* peak rapidly decreased, and a new absorption peak develops at 849 nm (1.46 eV) along with the development of a broad absorption feature around 0.5 eV. The intermediate-energy peak and the low-energy absorption could come from polarons, bipolarons, and polaron pairs, simultaneously. The peak at 849 nm could be assigned to excitation between the valence and conduction bands or a superposition of interband and valence-band-to-empty-level excitation. The low-energy broad absorption could be attributed to excitation from the valence band to the empty polaron/bipolaron orbitals in the gap.^21^ Such a variation of the absorption spectrum indicates that BDTTT forms polarons/bipolarons under the effect of the strong protic acid. As the trifluoroacetic acid is not an oxidizing agent and has extremely stable chemistry properties, it can only release protons in the solution (trifluoroacetic acid does not show a reduction in the cyclic voltammetry measurement, and it cannot oxide Fe^2+^ to Fe^3+^ as shown in Fig. S2.† At the same time, the mixture of BDTTT with sodium trifluoroacetate does not make any change on the absorption spectrum, as shown in Fig. S3†). This suggested that the proton adduction on the molecules is causing the change in the BDTTT solution absorption spectrum.

Since trifluoroacetic acid is easy to evaporate and other solid protonic acids such as 4-methylbenzenesulfonic acid are easy to crystallize, we fabricated the film samples with dodecylbenzenesulfonic acid (DDBSA). The pure BDTTT films show an absorption peak of π–π* at 422 nm, which is slightly shifted compared with the solution (414 nm). This may be attributed to the extended chains with longer conjugation length in the solid state. We don't observe any absorption shoulder like other oligothiophenes, which may suggest the stacking of the BDTTT is not very good due to a long sidechain. As compared to samples on a glass substrate, DDBSA-doped film shows a new absorption peak around 835 nm and broad absorption from 1200 nm to 2000 nm, which is similar to the observation in the solution (Fig. 1b). This suggests that BDTTT reacts with DDBSA almost instantly when the two materials come into contact. This makes sense because even in a diluted solution, BDTTT will react with the trifluoroacetic acid. Since DDBSA absorbs water from the air, the conductivity measurement could be influenced by the ion in the film. We annealed the film at 70 °C and measured the electrical conductivity at the same temperature. The electrical conductivity value of the film is about *ca.* 0.1 S cm^−1^ measured at 100 °C (to avoid the water uptake by the acid, as shown in Fig. S4 and Table S1†). This value is not high compared with state-of-art conducting polymers but it suggested BDTTT could be doped by proton and generated mobile charges. A higher conductivity could be expected through the morphological control of the BDTTT films.

To further confirm other protonic acids as dopants, we have fabricated the BDTTT on poly(4-styrene sulfonic acid) films. Compared with the samples on the glass substrate, the film on PSSH shows a new absorption peak around 850 nm and broad absorption from 1200 nm to 2000 nm, closed to the film doped using DDBSA. If we anneal the film on a hot plate at 100 °C for 10 min, the absorption at 850 nm and near Infrared range further enhanced, and the π–π* peak intensity decreased, suggesting the proton doping reaction is enhanced at a higher temperature (Fig. S5†).

**Fig. 2:**
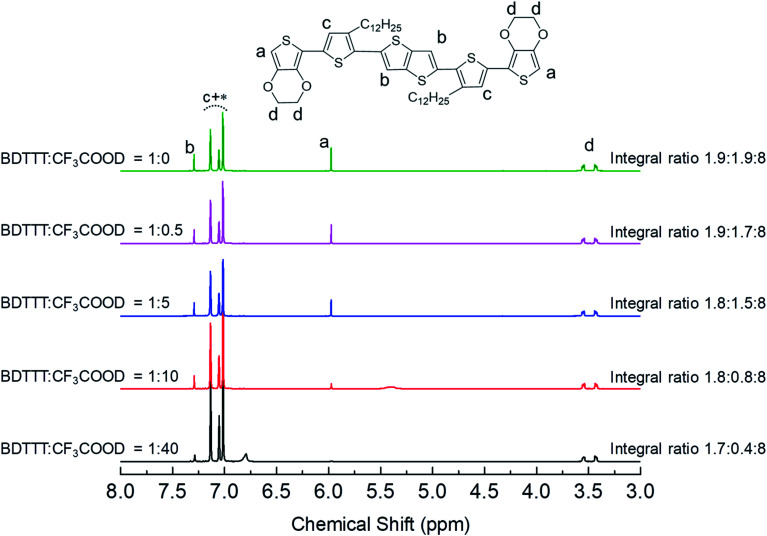
^1^H NMR spectra of BDTTT in toluene without and with addition of trifluoroacetic acid-d. Aromatic thiophene protons are indicated as c, which obscured by toluene signal which was indicated with an asterisk. a indicated the proton on thieno[3,2-*b*]thiophene, b indicated the proton at α-position of the end-thiophene, and d indicated the proton on ethylenedioxy. The integral ratio among b, a and d are shown on the graph.

As we mentioned in the introduction part, the advantage of BDTTT is its high solubility in various solvents, which allows us to confirm the doping sites on the molecule. We compared the ^1^H NMR spectra of BDTTT in Toluene with and without the addition of trifluoroacetic acid-d (CF_3_COOD), which is similar to the condition as Fig. 1a. Fig. 2 shows the NMR spectra of BDTTT in toluene-d8. In toluene, the chemical shift for the protons at the α-position of the end-thiophene is 5.97 ppm. The peaks of protons at the β-position of thieno[3,2-*b*]thiophene is 7.29 ppm. The thiophene protons obscured by the toluene signal in the range from 7.0–7.2 ppm. The –O–CH_2_– protons of EDOT units around 3.5 ppm as shown in Fig. 2.

**Fig. 3:**
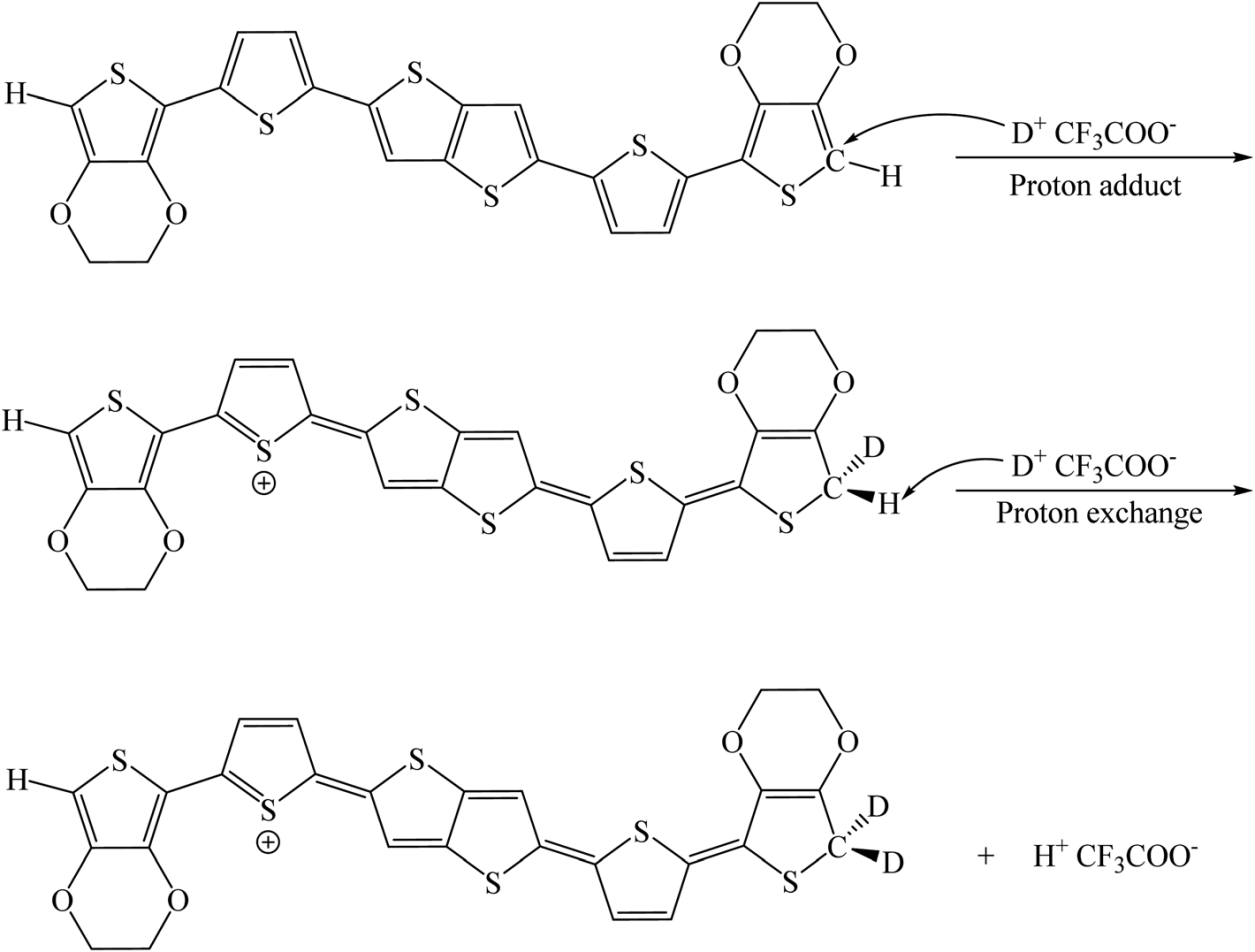
Possible doping process of BDTTT in CF_3_COOD.

**Fig. 4:**
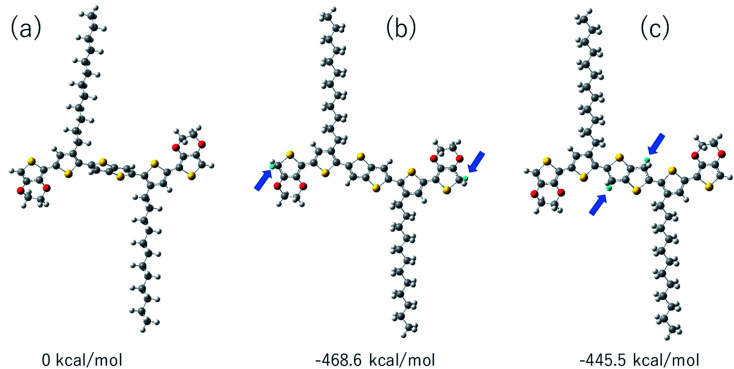
Ground-state geometry optimizations of: (a) BDTTT, adducts with H+ on (b) α-position of the end-thiophene, (c) the β-position of thieno[3,2-*b*]thiophene. Optimized structures were calculated using DFT at the B3LYP/6-31G(d) theory level. Relative energies are shown under each diagram. The H+ atoms are light-blue in color and are indicated by blue arrows.

We have compared the integral ratio of three proton signals b, a, d as shown in Fig. 2. Without the addition of CF_3_COOD, b : a : d is 1.9 : 1.9 : 8, which is closed to the number of protons in BDTTT. The molar ratio of BDTTT : CF_3_COOD is controlled from 1 : 0.1 to 1 : 40 during the addition of CF_3_COOD. As shown in Fig. 2, all the peaks are not shifted but the protons at the α-position of the end-thiophene is significantly reduced. This could be attributed to proton exchanging between α-hydrogen and deuterium ion of CF_3_COOD after doping, as shown in Fig. 3. At first, deuterium ion added to the α-position of BDTTT, makes the BDTTT proton-doped. The deuterium and hydrogen located on an α-position have the same chemical environment a high activity. The unreacted deuterium ion in solution could exchange with the deuterium or hydrogen on the α-position. Therefore, after the acid/BDTTT ratio increase, deuterium exchanges hydrogen, and the peak at 5.97 ppm in NMR spectra disappears. At the same time, there are new peaks appeared in the spectra (5.5 ppm for BDTTT : CF3COOD = 1 : 10 and 6.8 ppm for BDTTT : CF3COOD = 1 : 40). When the molar ratio of BDTTT : CF_3_COOD is 1 : 5, b : a : d is 1.8 : 1.5 : 8. The proton signal at the α-position of the end-thiophene is almost vanished when the molar ratio of BDTTT : CF_3_COOD increased to 1 : 40, implying that BDTTT is converted to its protonated form. Interestingly, the peaks of protons at the β-position of thieno[3,2-*b*] thiophene also started to decrease at high CF_3_COOD concentration. This suggested that the β-position of thieno[3,2-*b*] thiophene could also be protonated although the reactivity is lower than α-position of the end-thiophene. Density function theory (DFT) calculations were used to understand proton-BDTTT integration mechanisms. First, we optimized BDTTT at the B3LYP/6-31G(d) theory level as shown in Fig. 4a. The calculated band gap is 3.2 eV (HOMO 4.6 eV and LUMO 1.4 eV), corresponding to *ca.* 400 nm absorption edge. This is almost identical to the UV-vis-NIR result in the solution. We subsequently optimized adducts with protons independently bound to the α-position of the end-thiophene (Fig. 4b) and the β-position of thieno[3,2-*b*] thiophene (Fig. 4c). The relative energies determined from the optimized structures show that adduct formation at the α-position of thiophene is most favorable at a minimum of ∼469 kcal mol^−1^. Adducts with protons bound to the β-position of thieno[3,2-*b*] thiophene are a second favorable position with a minimum energy of ∼446 kcal mol^−1^ (Fig. 4c). (Please note that this is not Gibbs's free energy but the total energy of all the atoms and electrons after structure optimization). Interestingly, the doped molecule shows a much planar backbone. The dihedral angle formed between the plane of thieno[3,2-*b*]thiophene and thiophene ring changed from 136° to 179°. One possibility is that BDTTT forms a quinoid structure across the backbone after doping. We compared the C–C bond length and found the bond length alternation after proton doping (Fig. S6†), which coincides with the formation of quinoid structure. The calculation result is consistent with the NMR results and the knowledge of thiophene reactivity because the α-position is known to be activity and electron density at the α-position is high. We also carried out the time-dependent DFT calculation at the B3LYP/6-31G(d) theory level before and after proton doping.^21^ As shown in Fig. S7,† the calculated absorption peak of neutral BDTTT is around 440 nm which is closed to the absorption in the solution. After proton doping (1 charge, singlet state, *Q* = +1, *S* = 1/2), there is absorption peak around 800 nm. A triplet doubly charged charge carrier (polaron pair, *Q* = +2, *S* = 1) gives a board absorption from 1200 nm. This is closed to our experimental observation in the solution (Fig. 1a). On the other hand, please note that a singlet state is also possible for a doubly charged charge carrier, but the calculated absorption is very different from the experimental observation (Fig. S8†). Further investigation is needed to confirm the spin state after proton doping.^22,23^

Previous studies have shown that by 4-ethylbenzenesulfonic acid (EBSA) and (tridecafluoro-1,1,2,2-tetrahydrooctyl)trichlorosilane (FTS) can dope the semiconducting polymer poly(2,5-bis(3-tetradecylthiophen2-yl)thieno[3,2-*b*]thiophene) (PBTTT).^24,25^ It is important to note that the number of α positions on the end-thiophene is small because of the high molecular weight of PBTTT. Adducts with protons bound to the β-position of thieno[3,2-*b*] thiophene should be plausible. At the same time, it is important to note that, in the π-conjugated backbone, protons bound to carbon, have sp^3^ bonds, which break the conjugation.^26^ If we only consider a single molecule, the proton doping may yield lower carrier mobility. Recent studies show that electron transfer could occur from a neutral chain segment to a positively charged protonated segment, and therefore a high conductivity of proton doped PBTTT could be explained.^27,28^

## Conclusion

In conclusion, an organic semiconductor 2,5-bis(5-(2,3-dihydrothieno[3,4-*b*][1,4]dioxin-5-yl)-3-dodecylthiophen-2-yl)thieno[3,2-*b*]thiophene is designed and synthesized in this work. This molecule could be doped by protonic acid in both solution and solid-state, showing a broad absorption in the near near-infrared range corresponding to the absorption of polaron and bipolaron. The electrical conductivity was confirmed in the film samples. The adducts with protons bound at the end-thiophene α-position were confirmed by ^1^H Nuclear Magnetic Resonance spectra. This work highlighted that the use of protonated 3,4-ethylenedioxythiophene moieties as an end group to prepare organic conductors could be a promising approach.

## Experimental

### Chemicals

Ethanol, dodecylbenzenesulfonic acid (DDBSA), *p*-toluenesulfonic acid (TsOH), trifluoracetic acid (TFA) and toluene were purchased from FUJIFILM Wako Pure Chemical Co., Ltd. (Osaka, Japan). All other chemicals are from TCI Chemical Japan. All chemicals were used as received.

### Synthesis

#### 2,5-Bis(3-dodecylthiophen-2-yl)thieno[3,2-*b*]thiophene (3)

The toluene–EtOH–H_2_O (5 : 2 : 1, 24 mL) solution of 2,5-dibromothieno[3,2-*b*]thiophene (1) (870 mg, 2.92 mmol), 3-dodecyl-2-(4,4,5,5-tetramethyl-1,3,2-dioxaborolan-2-yl)thiophene (2) (2.43 g, 6.42 mmol) was blew by N_2_ gas to remove dissolved oxygen. Then K_2_CO_3_ (1.6 g, 12 mmol) and Pd(PPh_3_)_4_ (100 mg, 0.087 mmol) were added to the solution and heating under reflux for 24 h. After that, the solution was cooled down to room temperature, H_2_O was added for stopping the reaction and separate Toluene (organic layer). Organic phase was washed with saturated sodium chloride solution and dried over MgSO_4_, after that the solvent was removed by vacuum distillation, the obtained residue was washed with MeOH to obtain 2,5-bis(3-dodecylthiophen-2-yl)thieno[3,2-*b*]thiophene (3).

^1^H-NMR (CDCl_3_): *δ* 7.23 (s, 2H), 7.20 (d, *J* = 5.0 Hz, 2H), 6.95 (d, *J* = 5.0 Hz, 2H), 2.78 (t, *J* = 7.6 Hz, 4H), 1.64 (tt, *J* = 7.6, 7.6 Hz, 4H), 1.36–1.24 (m, 36H), 0.87 (t, *J* = 6.6 Hz, 6H).

#### 2,5-Bis(5-bromo-3-dodecylthiophen-2-yl)thieno[3,2-*b*]thiophene (4)

Compound 2,5-bis(3-dodecylthiophen-2-yl)thieno[3,2-*b*]thiophene (3) (1.60 g, 2.50 mmol) was dissolved in CHCl_3_ (32 mL), the solution was cooled in the cold bath, then DMF (20 mL) solution of *N*-bromosuccinimide (0.90 g, 5.0 mmol) was added dropwise. The mixed solution was stirred for 15 minutes in the cold bath, then the reaction mixture was concentrated under reduced pressure. The residue was added with MeOH, the solid obtained from filtration was 2,5-bis(5-bromo-3-dodecylthiophen-2-yl)thieno[3,2-*b*]thiophene (4).

^1^H-NMR (CDCl_3_): *δ* 7.16 (s, 2H), 6.90 (s, 2H), 2.69 (t, *J* = 7.3 Hz, 4H), 1.59 (m, 4H), 1.23 (br s, 36H), 0.85 (t, *J* = 6.6 Hz, 6H).

#### 2,5-Bis(5-(2,3-dihydrothieno[3,4-*b*][1,4]dioxin-5-yl)-3-dodecylthiophen-2-yl)thieno[3,2-*b*]thiophene

The DMF solution (10 mL) of 4 (400 mg, 0.501 mmol) was blew by N_2_ to remove dissolved oxygen. 5 (300 mg, 1.12 mmol), K_2_CO_3_ (300 mg, 2.17 mmol), Pd(PPh_3_)_4_ (30 mg, 0.026 mmol) was added to the solution, which was heated at 100 °C and stirred. The reaction undergoing was confirmed by ^1^H-NMR, and according to the condition, additional 5 (450 mg, 1.68 mmol) was added in the reaction by several times. After stirred for 24 hours, H_2_O was added for stopping the reaction and extract CH_2_Cl_2_. The obtained organic phase was washed by H_2_O, dried over MgSO_4_. Solvent was removed by vacuum distillation, the residue was separated by silica gel column chromatography then the EDOT–TTT–EDOT (225 mg, 0.244 mmol, 49%) was obtained.

^1^H-NMR (CDCl_3_): *δ* 7.24 (s, 2H), 7.05 (s, 2H), 6.24 (s, 2H), 4.36–4.34 (m, 4H), 4.26–4.24 (m, 4H), 2.77 (t, *J* = 7.8 Hz, 4H), 1.67 (m, 4H), 1.39–1.25 (m, 36H), 0.87 (t, *J* = 6.9 Hz, 6H).

^13^C-NMR (CDCl_3_): *δ* 141.9, 140.2, 139.1, 137.8, 137.6, 133.3, 129.2, 125.8, 117.5, 112.0, 97.1, 65.1, 64.6, 32.0, 30.7, 29.71, 29.69, 29.68, 29.6, 29.5, 29.43, 29.39, 22.7, 14.2 MALDI TOF-MS *m*/*z*: 920.31 (calc.), 920.33 (found).

### Film prepration

The glass substrate (2.5 cm × 2.5 cm) is cleaned by ultrasonication in detergent and rinsed with deionized water, then the substrate is cleaned by UV/ozone treatment. Before the film fabrication, the cleaned glass substrate is spin coated by HMDS at 3000 rpm for 40 seconds to make the substate surface hydrophobic. BDTTT is dissolved in Toluene at a concentration of 5 mg mL^−1^ (5.426 mM), DDBSA is dissolved in ethanol at a concentration of 54.26 mM. The BDTTT solution and the DDBSA solution are mixed in a volume ratio of 1 : 1, and the molar ratio of BDTTT and DDBSA is 1 : 10. The film is spin-coated from mix solution of BDTTT and DDBSA solution at 600 rpm for 30 seconds, then the films are heated at 70 °C to evaporate solvents and activate proton doping. The film thickness is around 30 nm.

### Characterization

The film thickness was measured by a surface profilometer (Sloan Dektak 3, Veeco). The conductivity was measured with a four-probe conductivity test meter (MCP-T600, Mitsubishi Chemical Corporation) and a semiconductor characterization system (Keithley 4200-SCS). The UV-Vis-NIR spectroscopy measurements were performed using a UV-Vis-NIR spectrometer (Solidspec-3700, Shimadzu). NMR spectra were record on an Bruker B400 system.

## Conflicts of interest

There are no conflicts to declare.

## Supplementary Material

RA-012-D2RA00216G-s001
