# Supplementary material for: Design and synthesis of proton-dopable organic semiconductors
Source: RSC Adv. 2022 Feb 28;12(11):6748–54. doi: 10.1039/d2ra00216g (PMC8981859; doi:10.1039/d2ra00216g)
Supplement: RA-012-D2RA00216G-s001 [file RA-012-D2RA00216G-s001.pdf]

Electronic Supplementary Material (ESI) for RSC Advances.
This journal is © The Royal Society of Chemistry 2022

Supporting Information

Design and Synthesis of Proton-dopable Organic Semiconductors

Chenzhu Yin[†,‡], Masakazu Mukaida[‡], Shohei Horike[‡], Kazuhiro Kirihara[‡], Shogo Yamane[§], Zhenya Zhang[†], and Qingshuo Wei [,‡]*

[†]Graduate School of Life and Environmental Sciences, University of Tsukuba, 1-1-1, Tennodai, Tsukuba, Ibaraki 305-8572, Japan

[‡]Nanomaterials Research Institute, Department of Materials and Chemistry, National Institute of Advanced Industrial Science and Technology, 1-1-1 Higashi, Tsukuba, Ibaraki 305-8565 Japan

[§]Research Institute for Sustainable Chemistry, Department of Materials and Chemistry, National Institute of Advanced Industrial Science and Technology (AIST), 1-1-1 Higashi, Tsukuba, Ibaraki 305-8565 Japan

E-mail: qingshuo.wei@aist.go.jp

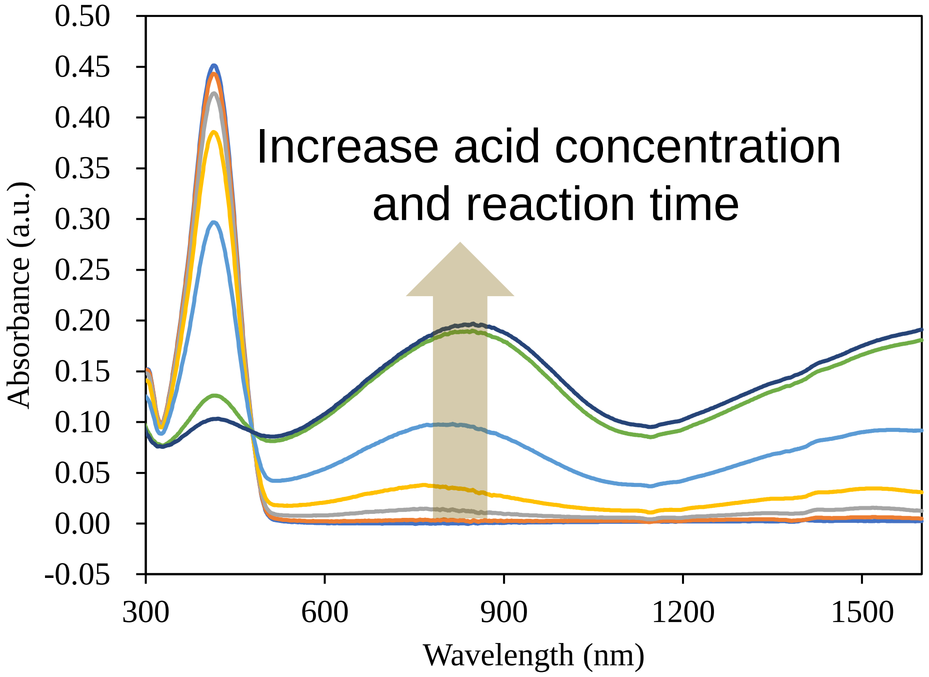

Figure S1. UV-Vis-NIR absorption spectra of pristine BDTTT solution (0.0125 mmol/L) and BDTTT solution with addition of TFA from 0.125 M to 0.375M

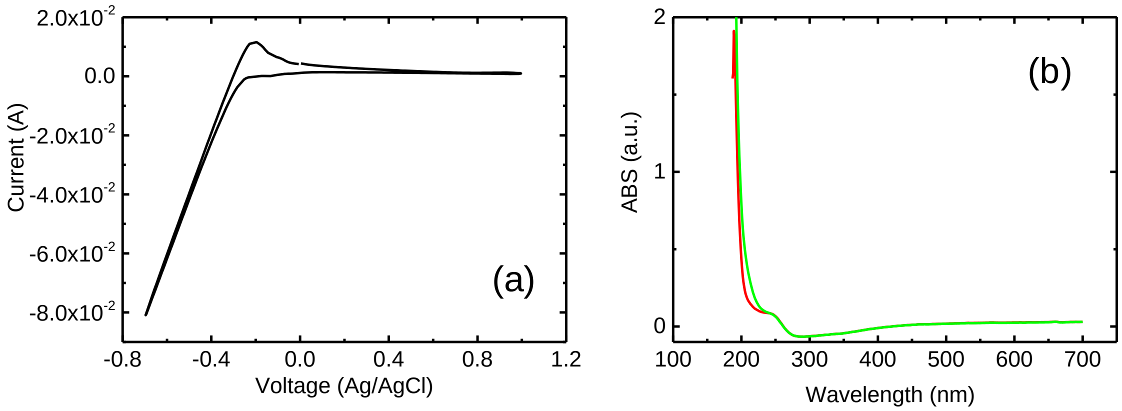

Figure S2. (a) CV of Pt electrodes at a scan rate of 100 mV/s in 1 M KCl and 10 mM CF_3COOH. (b) Absorption spectra of $FeCl_2$ solution without (Red line) and with CF_3COOH (Green line).

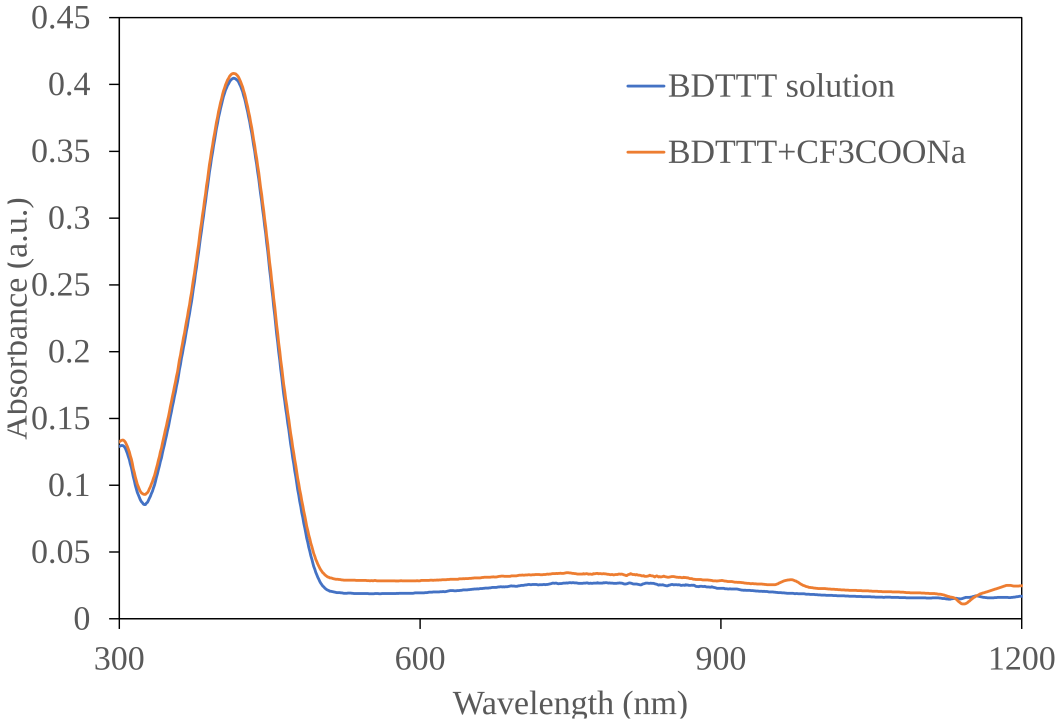

Figure S3. UV-Vis-NIR absorption spectra of pristine BDTTT solution and BDTTT solution with sodium trifluoroacetate.

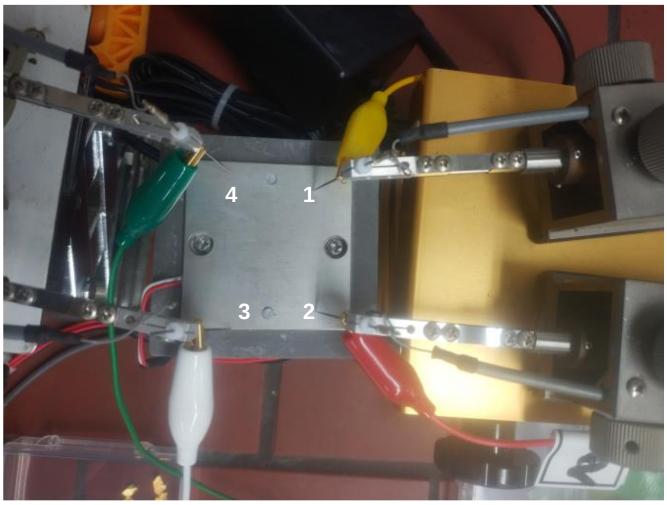

Figure S4. Picture of conductivity measurement set-up for a film sample

Current (A)	1.00E-06	2.00E-06	2.00E-06	2.00E-06	2.00E-06	2.00E-06
T(℃)	70	70	70	80	90	100
R34 (Ω)	295512.7	333606.7	169123.7	107517.4	145926.7	55141.7
R32 (Ω)	1525500	1316600	1888900	1843000	1457600	1163200
R43 (Ω)	195104.8	295127.3	415944.1	311197.5	235032	134049.1
R41 (Ω)	1042500	1430900	1505200	2406400	3343500	3829700
R14 (Ω)	876996.3	466443.8	460012.9	262749.6	88447.6	35014.2
R12 (Ω)	16507.6	9631.6	30732.6	265659.9	458801.9	533531.3
R23 (Ω)	1121100	1542400	1572000	1500500	1120200	656230.3
R21 (Ω)	355480.2	402888.9	362999	429025.4	151202.4	164785.7
R=AVG (Ω)	6.79E+05	7.25E+05	8.01E+05	8.91E+05	8.75E+05	8.21E+05
Rv (Ω)	2.16E+05	2.60E+05	2.45E+05	2.78E+05	2.48E+05	2.22E+05
Rh (Ω)	1.14E+06	1.19E+06	1.36E+06	1.50E+06	1.50E+06	1.42E+06
Rs=ΠR/ln2	3.08E+06	3.28E+06	3.63E+06	4.04E+06	3.97E+06	3.72E+06

Table S1. The raw data of the condcutivity meaurement of DDBSA-BDTTT film at different temperature

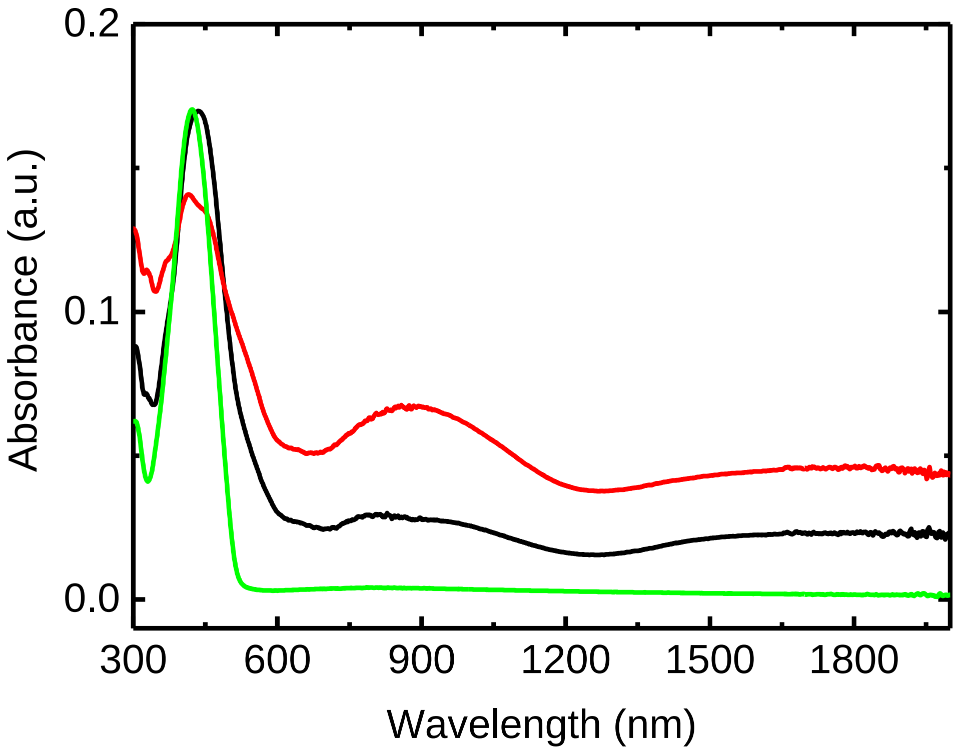

Figure S5. Absorption spectra of BDTTT film on glass (Green line), PSSH (Black line) and PSSH annealed at 150 °C from 10 mins.

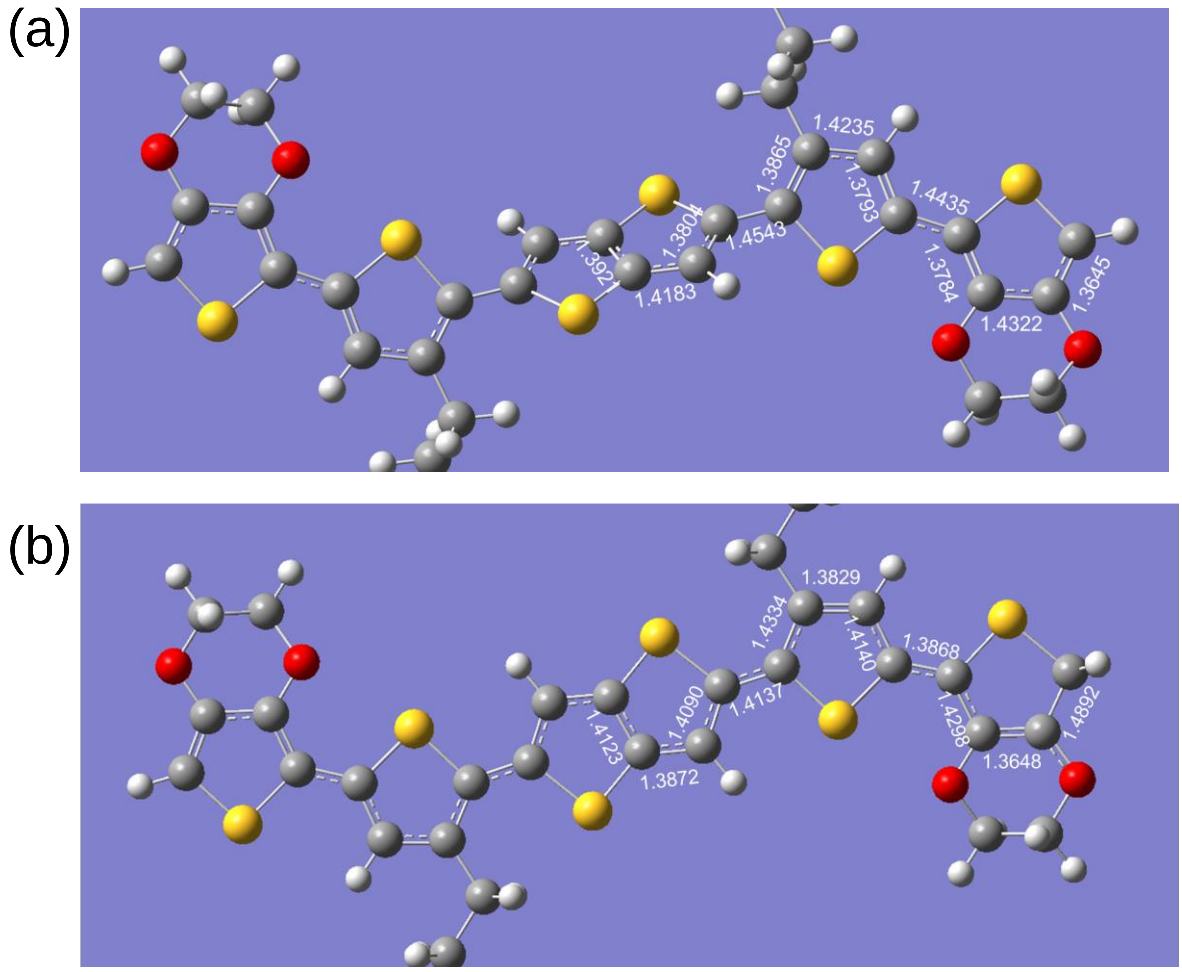

Figure S6. Calculated C-C bond length before (a) and after (b) proton doping.

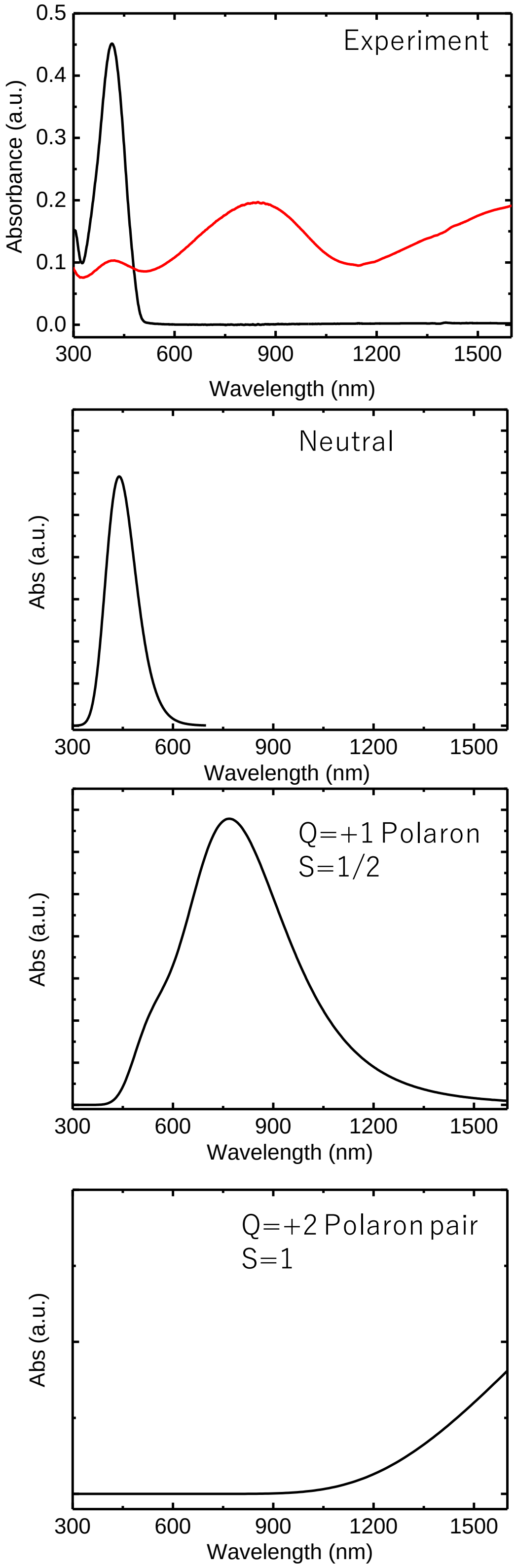

Figure S7. Experimental and calculated absorption spectra of BDTTT with the different doping levels (neutral, polaron, and polaron pair).

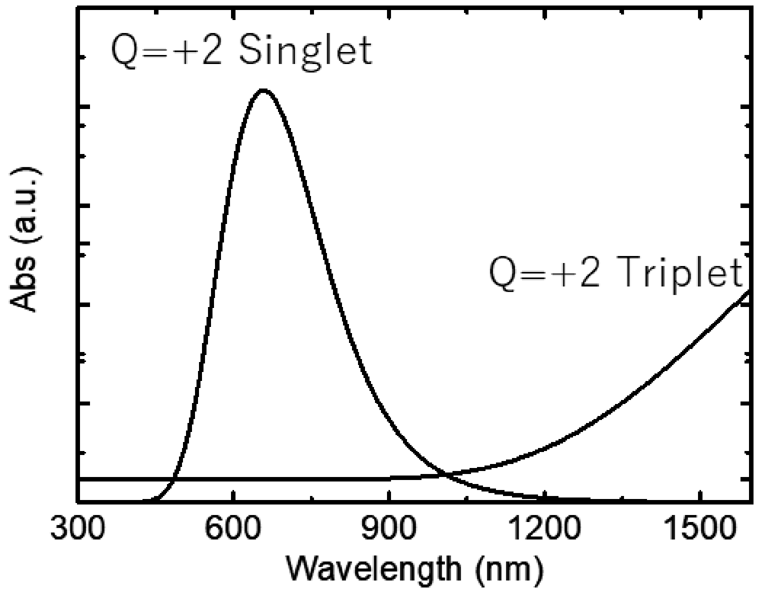

Figure S8. Comparison of the calculated absorption spectra of BDTTT after doping (Q = +2) with singlet and triplet state.

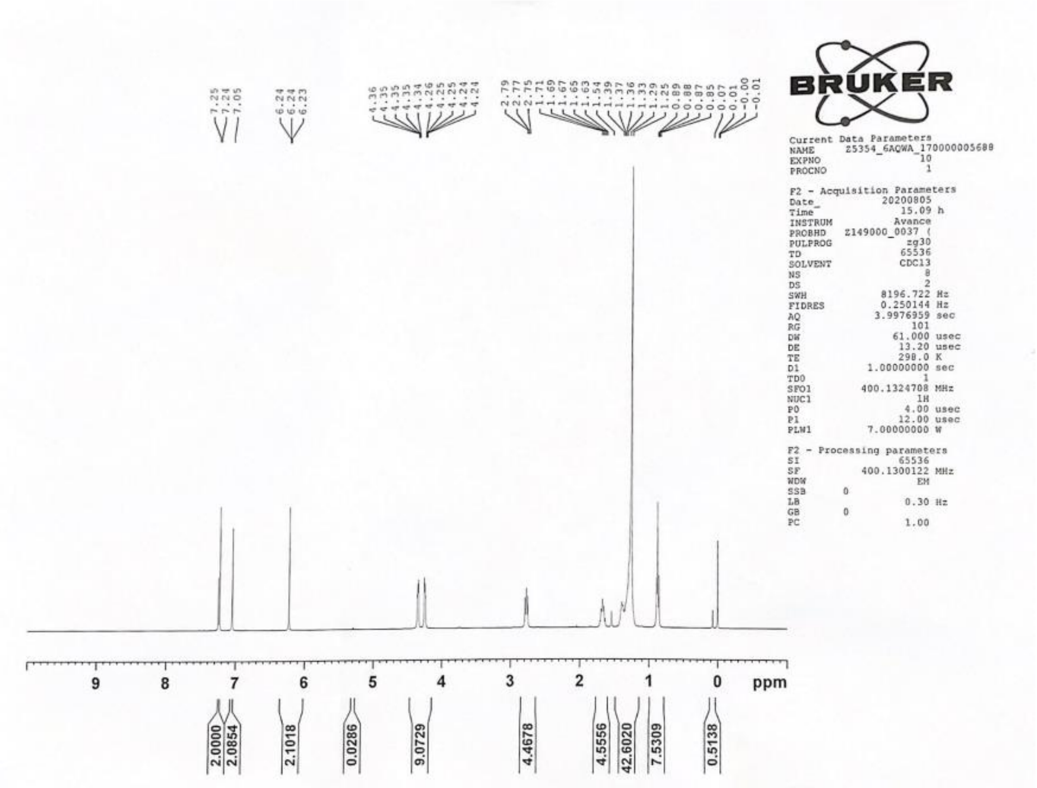

Figure S9. ^{1}H NMR spectra of BDTTT in Chloroform

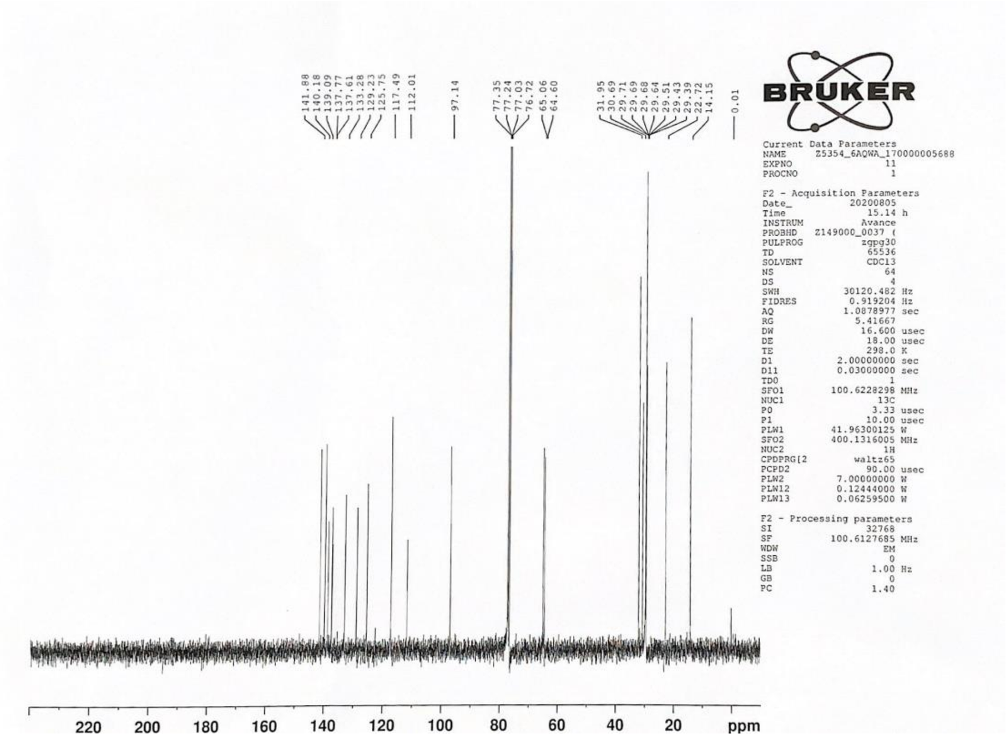

Figure S10. ^{13}C NMR spectra of BDTTT in Chloroform

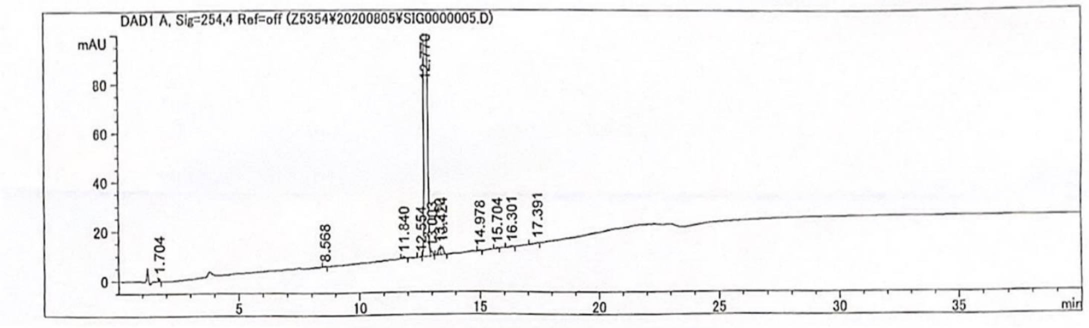

ピーク #	RT [min]	タイプ	ピーク幅 [min]	面積 [mAU*s]	高さ [mAU]	面積 %
1	1.704	BB	0.0428	4.03142	1.47881	0.0926
2	8.568	BB	0.0874	1.45030	2.58884e-1	0.0333
3	11.840	BB	0.1078	5.57893	7.95532e-1	0.1281
4	12.554	BV	0.0741	1.60789	3.12049e-1	0.0369
5	12.770	VV	0.0892	4281.46191	754.20013	98.3133
6	13.003	VB	0.1005	11.77964	1.84472	0.2705
7	13.310	BV	0.0848	17.08046	3.02987	0.3922
8	13.424	VB	0.1013	22.17272	3.26206	0.5091
9	14.978	BB	0.0909	2.30980	3.91099e-1	0.0530
10	15.704	BB	0.0746	1.20806	2.53591e-1	0.0277
11	16.301	BB	0.1323	4.27671	4.32927e-1	0.0982
12	17.391	BB	0.1091	1.95936	2.53606e-1	0.0450
トータル：				4354.91719	766.51328	

Figure S11. Liquid Chromatogram of of BDTTT

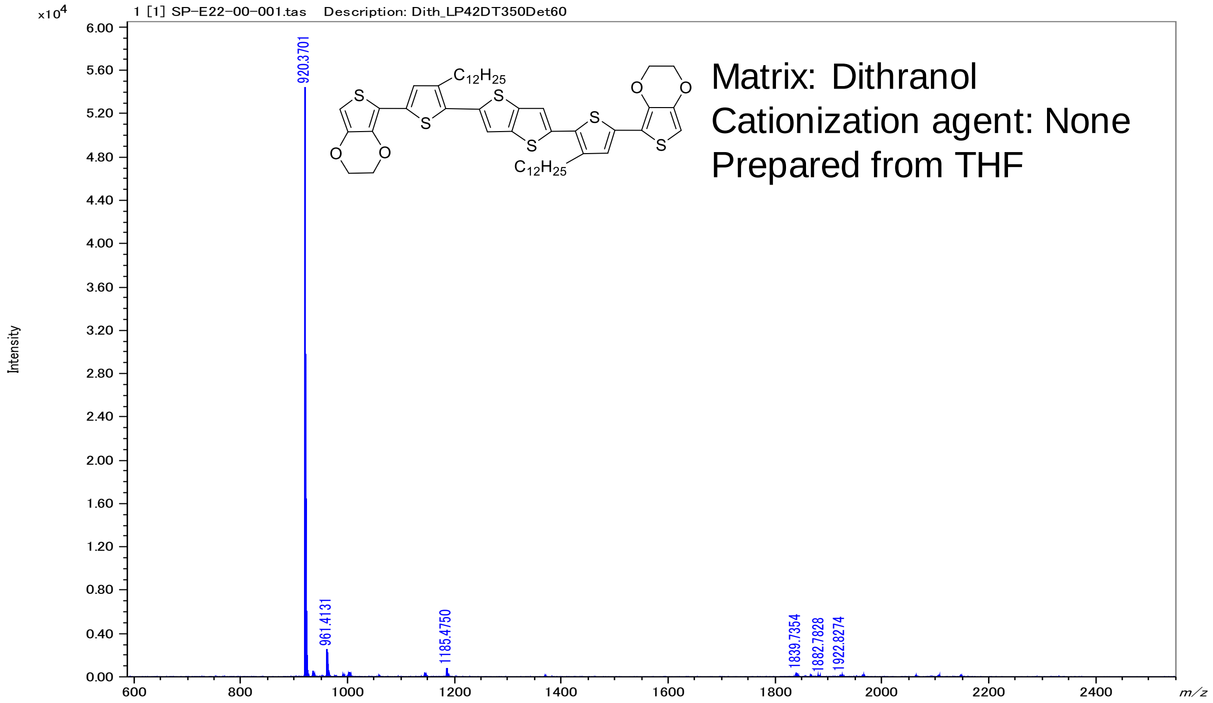

Figure S12. Time of Flight Mass Spectrometry of BDTTT
